# Improving the Quality of Nursing Care Through Clinical Alarm Management in Intensive Care Units: An Integrative Review

**DOI:** 10.3390/healthcare14172720

**Published:** 2026-08-26

**Authors:** Valter Ferreira, Nuno Carrajola, Maria José Catalão

**Affiliations:** 1 Hospital Lusíadas Albufeira, Lusíadas Saúde, Largo da Correeira-Montechoro, 8200-112 Albufeira, Portugal; 2 Department of Health Sciences and Technologies, School of Health, Portalegre Polytechnic University, Campus Politécnico 10, 7300-555 Portalegre, Portugal; nuno.carrajola@ipportalegre.pt; 3 CARE-Research Center on Health and Social Sciences, Portalegre Polytechnic University, 7300-555 Portalegre, Portugal

**Keywords:** clinical alarms, physiologic monitoring, patient safety, critical care nursing, quality of health care, intensive care units

## Abstract

**Highlights:**

**What are the main findings?**

- Five key domains of nursing interventions were identified for improving clinical alarm management in intensive care units.
- Multifaceted alarm management strategies were associated with reductions in nonactionable alarms and with safer patient monitoring.

**What are the implications of the main findings?**

- Evidence-based alarm management programs can strengthen patient safety and improve the quality of critical care.
- Nurses play a central role in implementing effective alarm management through clinical judgment, education, and evidence-based practice.

**Abstract:**

**Background/Objectives**: Clinical alarm management in continuous patient monitoring systems remains a major challenge in intensive care units (ICUs). The high frequency of nonactionable alarms contributes to alarm fatigue among healthcare professionals, potentially compromising patient safety, clinical surveillance, and the quality of nursing care. As nurses play a central role in monitoring critically ill patients, evidence-based alarm management strategies are essential to improve the quality of care and patient outcomes. This review aimed to synthesize the scientific evidence on nursing interventions for managing clinical alarms in continuous monitoring systems and their contribution to patient safety and quality of care among critically ill patients. **Methods**: An integrative literature review was conducted following the methodological framework proposed by Whittemore and Knafl. A comprehensive literature search was performed in CINAHL Plus with Full Text, MEDLINE with Full Text, Supplemental Index, Complementary Index, Academic Search Index, the Directory of Open Access Journals (via EBSCOhost^®^), PubMed, Scopus, and ScienceDirect. Studies published between 2020 and 2025 in English, Portuguese, or Spanish were eligible for inclusion. Methodological quality was assessed using the Joanna Briggs Institute critical appraisal tools. Nine studies met the eligibility criteria and were included in the final synthesis. **Results**: The identified interventions were grouped into five main domains: alarm parameter customization, nurse education and training, implementation of structured protocols and alarm management bundles, optimization of monitoring devices and sensors, and interventions addressing human and organizational factors. The evidence indicated that multifaceted and integrated interventions were associated with reductions in nonactionable alarms and improvements in clinical responses to alarms. However, their effectiveness in reducing alarm fatigue appeared to depend on organizational culture, staff engagement, and contextual factors. **Conclusions**: Effective alarm management requires the consistent implementation of evidence-based nursing interventions. Nurses play a pivotal role in promoting patient safety through clinical expertise, technological competence, and adherence to structured alarm management practices. Strengthening alarm management strategies may contribute to improved quality of care, safer care delivery, and better outcomes for critically ill patients.

## 1. Introduction

Patient safety is a fundamental dimension of healthcare quality, particularly in highly complex settings such as intensive care units (ICUs), where critically ill patients require continuous surveillance and rapid clinical decision-making. Ensuring safe care in these environments has become an international priority because of the increasing complexity of healthcare delivery, technological dependence, and the recognition that preventable adverse events remain a major challenge for healthcare systems worldwide [1,2].

Continuous physiological monitoring is indispensable in ICUs because it enables the early detection of clinical deterioration and supports timely therapeutic interventions. Advances in monitoring technologies have substantially improved patient surveillance and contributed to safer clinical practice. However, the widespread implementation of monitoring systems has also resulted in a considerable increase in the number of clinical alarms, creating an important patient safety paradox: technologies designed to enhance safety may themselves become a source of risk when alarms are excessive or poorly managed [3,4,5]. Evidence consistently demonstrates that a substantial proportion of alarms generated by physiological monitors are nonactionable or clinically irrelevant, with reported estimates ranging from approximately 72% to 99% in earlier observational studies and up to 94% in more recent evidence syntheses [3,6,7]. This excessive alarm burden contributes to increased environmental noise, workflow interruptions, and cognitive overload among healthcare professionals [8]. Persistent exposure to this alarm burden can lead to alarm fatigue, a phenomenon characterized by progressive desensitization to alarm signals, delayed responses, inappropriate silencing of alarms, and an increased risk of missing clinically significant events [4,6,9]. Alarm fatigue remains a major patient safety challenge in technology-intensive healthcare environments and reflects the interaction of technological, organizational, and human factors rather than alarm frequency alone [5].

The importance of safe alarm management has been recognized internationally. Following reports of serious adverse events associated with failures to recognize or respond appropriately to medical device alarms, The Joint Commission incorporated alarm safety into the National Patient Safety Goals, establishing the improvement of clinical alarm systems as an ongoing patient safety priority [10,11,12]. Likewise, the Global Patient Safety Action Plan 2021–2030 published by the World Health Organization (WHO) identifies the safe use of health technologies, the promotion of a patient safety culture, and systematic approaches to clinical risk management as strategic priorities for healthcare systems worldwide [1]. The principles outlined in the WHO Global Patient Safety Action Plan have also informed European patient safety priorities, with improvements in safety culture and quality of care and the prevention of avoidable harm remaining central objectives [1]. These priorities are also reflected in national policies across Europe. In Portugal, for example, the National Plan for Patient Safety 2021–2026 establishes strategic actions aimed at strengthening patient safety culture, preventing adverse events, improving clinical risk management, and promoting safer clinical practices [13].

In this context, nurses play a pivotal role in clinical alarm management because they are primarily responsible for continuous patient surveillance, alarm parameter configuration, and the interpretation of physiological monitoring data. Their proximity to critically ill patients places them in a unique position to recognize clinically significant changes and respond promptly to alarms. Nevertheless, nurses are also among the professionals most affected by alarm fatigue, frequently experiencing cognitive overload, stress, and reduced attention, all of which may compromise clinical judgment and patient safety [14,15]. Nurses’ clinical judgment, technological competence, adaptive coping strategies, and organizational support are fundamental to effective alarm management and to mitigating the effects of alarm fatigue in intensive care environments [9].

To address these challenges, multifaceted alarm management strategies have been proposed, including the individualization of alarm parameters, staff education, structured institutional protocols, optimization of monitoring devices, and organizational interventions aimed at reducing nonactionable alarms [16,17]. Although research in this field has expanded considerably, the available evidence regarding nursing interventions for clinical alarm management in ICUs remains fragmented across different study designs and clinical contexts. Consequently, a synthesis of the existing evidence is needed to identify the most effective nursing strategies for improving alarm management, reducing alarm fatigue, and enhancing patient safety. In light of these challenges, this integrative review aimed to synthesize and critically appraise the available evidence on nursing interventions for managing clinical alarms generated by continuous physiological monitoring systems and the contribution of these interventions to patient safety and the quality of care in ICUs.

## 2. Materials and Methods

### 2.1. Study Design

An integrative literature review was conducted following the methodological framework proposed by Whittemore and Knafl [17]. This approach enables the integration of evidence derived from diverse methodological designs, providing an understanding of complex healthcare phenomena and supporting evidence-based clinical practice. The review followed the five stages described by Whittemore and Knafl [17]: problem identification, literature search, data evaluation, data analysis, and presentation of the findings. The review protocol was registered with the Open Science Framework (OSF) (https://osf.io/ajv9t/overview; accessed on 20 August 2026).

This review sought to answer the following research question: Which nursing interventions for clinical alarm management during continuous physiological monitoring contribute to patient safety in intensive care units?

### 2.2. Search Strategy

A comprehensive literature search was conducted between October and December 2025 using the EBSCOhost^®^ platform, including CINAHL Plus with Full Text, MEDLINE with Full Text, Supplemental Index, Complementary Index, Academic Search Index, and the Directory of Open Access Journals (DOAJ). Additional searches were conducted in PubMed, Scopus, and ScienceDirect.

The search strategy combined controlled vocabulary terms, including Medical Subject Headings (MeSH) where applicable, and free-text terms related to the review topic, including *monitoring, physiologic, clinical alarms, patient safety, safety management, critical care, intensive care units, nursing care,* and *critical care nursing*. Boolean operators (AND/OR) were used to optimize the search strategy.

The complete search string was as follows: “monitoring, physiologic” AND “clinical alarms” AND (“patient safety” OR “safety management”) AND (“critical care” OR “intensive care units”) AND (“nursing care” OR “critical care nursing”).

The search strategy was adapted according to the indexing characteristics and search functionalities of each database. In EBSCOhost^®^, the option to expand equivalent terms was activated, whereas in Scopus, the search was limited to the title, abstract, and keyword fields within the nursing subject area. The same concepts and Boolean structure were maintained across all databases.

### 2.3. Eligibility Criteria

Eligibility criteria were predefined according to the review objective and are summarized in Table 1. Studies employing diverse methodological designs were considered eligible, consistent with the integrative review methodology and the aim of providing a comprehensive understanding of the available evidence.

Full-text availability was defined as access to the complete article through the databases and electronic resources available to the reviewers. All 13 reports sought for retrieval following title and abstract screening were successfully retrieved and assessed for eligibility; therefore, no reports were excluded at the retrieval stage because the full text was unavailable.

### 2.4. Study Selection

Following the application of the predefined eligibility filters during the database searches, including publication year (2020–2025), language (English, Portuguese, or Spanish), full-text availability, peer-reviewed publications, and database-specific search limits (e.g., the nursing subject area in Scopus and equivalent term expansion in EBSCOhost^®^), a total of 38 records were identified across the selected information sources. All retrieved references were exported to Zotero (version 9.0.5), which was used for reference management and duplicate identification. Following the removal of 11 duplicate records, 27 articles underwent title and abstract screening by two independent reviewers. Fourteen studies were excluded at this stage because they did not meet the eligibility criteria. The remaining 13 full-text articles were independently assessed by the same reviewers. Three studies were excluded because they did not address nursing interventions or nursing practice related to clinical alarm management within the scope of the review, and one scoping review was excluded because it substantially overlapped with the included primary studies. Any disagreements during the study selection process were resolved through discussion until consensus was reached. Consequently, nine studies were included in the final evidence synthesis.

### 2.5. Methodological Quality Assessment

Methodological quality was independently assessed by two reviewers using the appropriate Joanna Briggs Institute (JBI) Critical Appraisal Tools for each study design (Appendix A, Table A1). The JBI Critical Appraisal Checklist for Systematic Reviews and Research Syntheses was applied to the integrative review [18], the JBI Checklist for Quasi-Experimental Studies to quasi-experimental and quality improvement studies [19], the JBI Checklist for Analytical Cross-Sectional Studies to cross-sectional studies [20], and the JBI Checklist for Qualitative Research to qualitative studies [21]. Each study was appraised at the item level using the response categories provided by the relevant JBI Critical Appraisal Tool (“Yes”, “No”, “Unclear”, or “Not applicable”, as appropriate). To avoid implying numerical comparability across different study designs and JBI tools, the appraisal judgments were not converted into numerical or percentage scores, and “Not applicable” responses were retained as such rather than assigned a numerical value. The methodological appraisal was used to identify methodological strengths and limitations and to support the critical interpretation of the evidence; it did not determine study inclusion or exclusion. Any disagreements between the two reviewers were resolved through discussion until consensus was reached. Detailed item-level appraisal results are presented in Appendix A, Table A1.

### 2.6. Data Extraction and Synthesis

**Figure 1:**
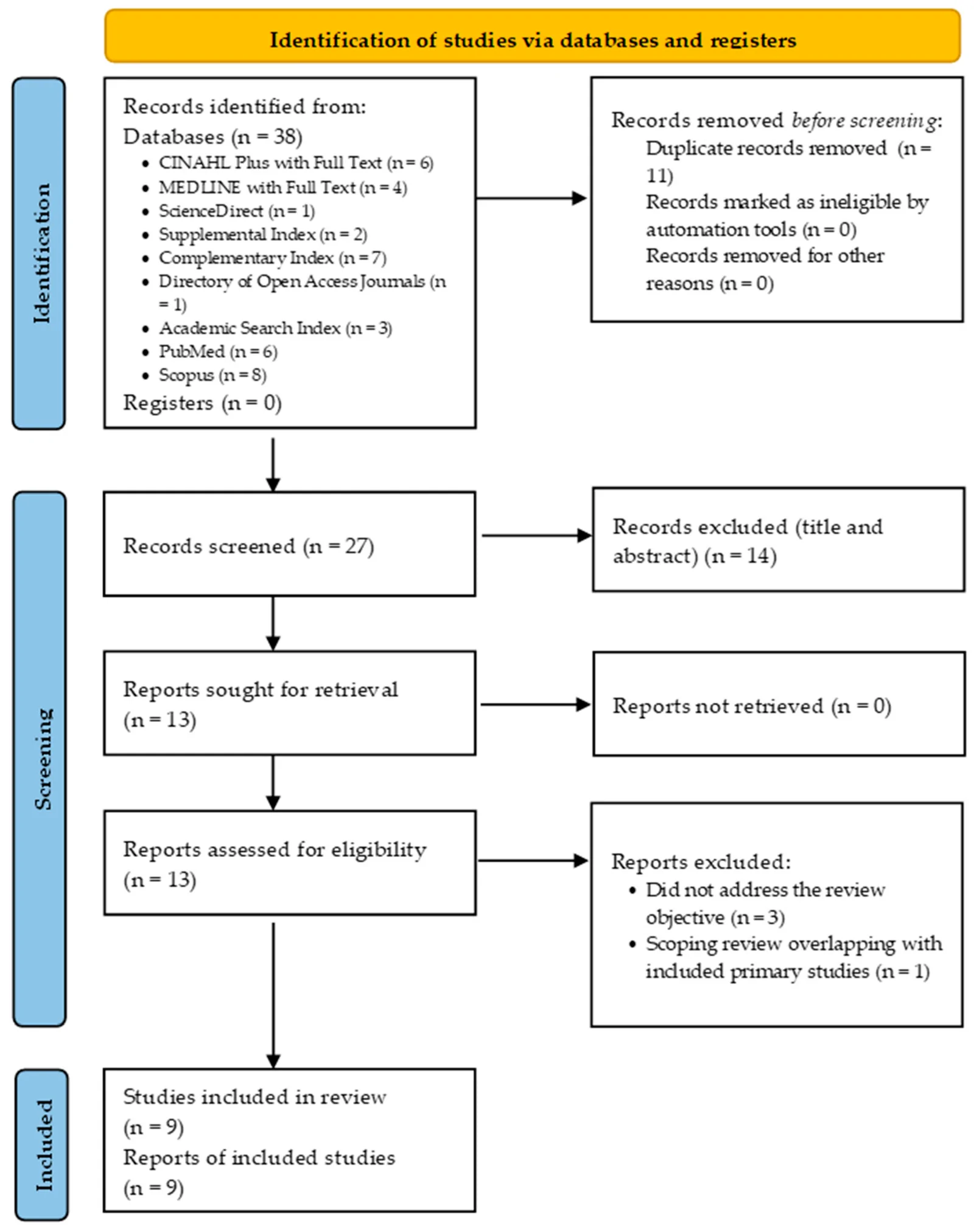
Flow diagram of the study selection process in accordance with PRISMA 2020.

The study selection process reported in accordance with the Preferred Reporting Items for Systematic Reviews and Meta-Analyses (PRISMA 2020) statement and is presented in the PRISMA flow diagram (Figure 1).

Data were systematically extracted using a standardized data extraction form that included study characteristics, methodological design, nursing interventions, and main findings. The extracted nursing interventions were compared across the included studies and grouped according to their conceptual similarities. Through an iterative process of data comparison and discussion between the reviewers, five thematic domains were identified and used to organize the findings.

## 3. Results

Following the study selection process, nine studies met the eligibility criteria and were included in this integrative review. The included studies were published between 2021 and 2025 and comprised a range of methodological designs, including quality improvement projects, quasi-experimental, cross-sectional, and qualitative studies, as well as one integrative review. Most studies were conducted in the United States, with additional evidence originating from China, Iran, South Korea, and Turkey. Overall, the included studies investigated nursing interventions aimed at improving clinical alarm management in intensive care settings.

The methodological quality of the included studies was assessed using the appropriate JBI Critical Appraisal Tools for each study design. Given the methodological diversity of the included studies and the use of different JBI appraisal tools, the appraisal findings were interpreted at the item level rather than converted into numerical or percentage scores for comparison across study designs. The appraisal identified methodological strengths and limitations in the included studies, which were considered when interpreting the findings of the review. All nine studies were retained in the final evidence synthesis, regardless of their individual appraisal findings. Detailed item-level JBI appraisal results are presented in Appendix A, Table A1. Table 2 summarizes the characteristics of the included studies, including the authors, year of publication, country, study design, setting or sample, and study aim.

The analysis of the included studies identified five major domains related to nursing interventions and clinical alarm management in ICUs: (1) individualization of alarm parameters, (2) nurse education and training, (3) structured alarm management protocols and strategies, (4) optimization of monitoring devices and sensors, and (5) human and organizational factors influencing alarm management. Across the included studies, interventions were predominantly multifaceted, combining educational, technological, and organizational strategies to reduce nonactionable alarms and improve patient safety. Although the specific interventions varied across studies, there was broad agreement that effective alarm management requires an integrated approach involving both technological optimization and nursing clinical decision-making. Table 3 presents the nursing interventions and associated factors identified in the included studies according to the five thematic domains and summarizes their key findings.

## 4. Discussion

Effective clinical alarm management in ICUs is a multifaceted process that extends beyond the optimization of monitoring technology alone. The findings indicate that reducing nonactionable alarms requires the integration of patient-centered individualization of alarm parameters, continuous nurse education, structured organizational protocols, optimization of monitoring devices, and consideration of the human and organizational factors that influence clinical decision-making. Collectively, these interventions reinforce the pivotal role of nurses in effective clinical alarm management. From a quality-of-care perspective, these findings can be interpreted through Donabedian’s Structure–Process–Outcome framework [29]. Donabedian’s broader conceptualization of quality of care also emphasizes dimensions such as effectiveness, efficiency, optimality, acceptability, legitimacy, and equity; however, these dimensions could not be comprehensively evaluated because they were not systematically assessed in the included studies. Although this framework was not used to derive the five thematic domains, it provides a useful conceptual lens for interpreting them. Structural elements include monitoring technologies, equipment, staffing, and organizational support; process elements encompass alarm parameter individualization, standardized protocols, education, teamwork, and nurses’ alarm management practices; and the outcomes evaluated in the included studies primarily included alarm burden, alarm fatigue, and nurses’ knowledge or competence. Importantly, direct patient-level clinical outcomes were rarely assessed; therefore, the findings should not be interpreted as demonstrating direct improvements in patient safety or patient outcomes.

Although the included studies consistently support the implementation of multifaceted alarm management strategies, most of the available evidence derives from quality improvement projects and observational research conducted in specific clinical settings. Consequently, these findings should be generalized with caution.

### 4.1. Individualization of Alarm Parameters

Tailoring alarm parameters to individual patient characteristics represents an important component of effective alarm management. Rather than relying exclusively on default limits, individualized settings allow monitoring parameters to reflect patients’ physiological condition and evolving clinical status. This approach integrates monitoring technology with nurses’ clinical judgment and continuous patient assessment [4,27] and can be incorporated into broader institutional alarm management strategies [16].

Default alarm thresholds may generate high numbers of nonactionable alarms in critically ill patients, contributing to alarm burden and alarm fatigue [3,6]. Within the included evidence, tailoring alarm limits to individual patient characteristics was associated with changes in alarm burden and perceived alarm relevance [27,28]. Clemenson and Tellson {25], for example, reported that adjusting heart rate and respiratory rate limits by approximately ±15% reduced nonactionable alarms by up to 43%. Qualitative evidence further suggests that nurses perceive continuous reassessment of alarm parameters as part of clinical reasoning and patient surveillance [27], consistent with recent evidence describing individualization as a dynamic clinical process rather than a one-time technical adjustment [9].

These findings should nevertheless be interpreted cautiously because most studies evaluating alarm individualization used quasi-experimental or quality improvement designs, limiting causal inference. Moreover, changes in alarm frequency should not be considered equivalent to improvements in patient safety or clinical outcomes, which were rarely assessed directly. Indeed, one included study reported an increase in overall alarm frequency following parameter individualization [30], reinforcing the need to consider alarm relevance and clinical response alongside alarm counts [31]. This cautious interpretation is consistent with evidence from a systematic review and meta-analysis showing substantial heterogeneity across alarm management interventions and only a small overall effect on alarm frequency [32]. Alarm parameter individualization should therefore be understood as a dynamic clinical process embedded with broader organizational, educational, and monitoring strategies rather than as an isolated technical intervention.

### 4.2. Nurse Education and Training

Nurse education and continuous professional development emerged as important components of alarm management. Effective practice requires nurses to interpret physiological data, recognize clinically significant alarms, and integrate monitoring information into clinical decision-making [24,28]. Structured educational interventions have been associated with improvements in knowledge, confidence, alarm management practices, and self-reported alarm fatigue [31]. Multifaceted programs combining education with alarm parameter individualization, device optimization, and standardized practices, such as the CEASE bundle, have also demonstrated improvements in nurses’ competencies and alarm-related process outcomes [22,24]. However, because these interventions include multiple components, the independent contribution of education cannot be established.

Importantly, improvements in knowledge and competence do not necessarily translate into reductions in alarm frequency or improvements in patient outcomes. The effectiveness of educational interventions is influenced by organizational factors such as workload, staffing, leadership support, and opportunities to reinforce competencies in clinical practice [28]. As monitoring systems become increasingly sophisticated, continuing education should therefore combine technological literacy with clinical reasoning and competency development [33] and be integrated into broader organizational alarm management strategies.

### 4.3. Structured Alarm Management Protocols and Strategies

Structured alarm management protocols provide an organizational mechanism for integrating technological optimization, evidence-based clinical practice, staff education, and organizational support. Across the reviewed evidence, multifaceted strategies, including the CEASE protocol and interventions informed by the American Association of Critical-Care Nurses recommendations, were associated with reductions in nonactionable alarms and improvements in nurses’ competencies and alarm management processes [15,16,23,24]. These findings support systematic institutional approaches rather than isolated behavioral or technical interventions [34].

However, alarm frequency alone provides an incomplete measure of intervention effectiveness. Evaluation should consider alarm relevance, alarm fatigue, response practices, workflow integration, and, where available, direct patient-level outcomes [35]. In the present review, evidence on direct patient-level outcomes remained limited.

Sustained implementation also requires organizational commitment through multidisciplinary collaboration, leadership support, regular audits, and continuous quality improvement processes [12,34]. Accordingly, structured alarm management protocols should be regarded as dynamic systems that require ongoing evaluation and adaptation to local clinical and technological contexts.

### 4.4. Optimization of Monitoring Devices and Sensors

Optimization of monitoring devices and sensors is an important component of alarm management because technical artifacts and poor signal acquisition contribute to nonactionable alarms [6,22,24,34]. Relatively simple interventions, including appropriate skin preparation, electrode placement, and routine electrode replacement, have been associated with improved signal quality and reductions in technical alarms [22,23]. However, most evidence derives from single-center or specialized ICU settings, limiting generalizability to different monitoring technologies and clinical environments.

Monitoring performance also depends on appropriate equipment maintenance and the integration of technology into clinical workflows. Intelligent monitoring and notification systems may support alarm prioritization [15], but technological solutions remain dependent on reliable physiological signals and appropriate clinical interpretation [5]. Moreover, reductions in technical alarms do not necessarily translate into corresponding reductions in alarm fatigue or improved patient outcomes, as these are influenced by interacting technological, cognitive, environmental, and organizational factors [23]. Device optimization should therefore form part of a broader alarm management strategy rather than be considered a standalone intervention.

### 4.5. Human and Organizational Factors in Alarm Management

Human factors substantially influence how clinical alarms are interpreted, prioritized, and acted upon. Repeated exposure to nonactionable alarms may contribute to cognitive overload, desensitization, and alarm fatigue [8,26], while nurses’ experience, clinical judgment, and confidence in alarm reliability influence alarm management practices [8,9,26]. Nurses also report adaptive strategies including alarm parameter individualization, anticipation of predictable alarms, teamwork, emotional self-regulation, and clinical prioritization [27,36]. These findings support an understanding of alarm fatigue as a sociotechnical phenomenon rather than simply a consequence of alarm frequency [5]. This multifactorial interpretation is also consistent with systematic review evidence showing that alarm fatigue among nurses is associated with interacting individual, alarm-related, and organizational factors [37].

Organizational conditions, including workload, staffing, interruptions, leadership support, teamwork, and the clinical environment, may further influence nurses’ ability to manage alarms effectively [5,8,26,30,38]. Sustainable alarm management therefore requires attention to both technological and organizational conditions, supported by multidisciplinary collaboration and continuous professional development. Such strategies may improve alarm management processes and the clinical work environment; however, evidence of direct effects on patient safety outcomes remains limited among the included studies.

### 4.6. Clinical Implications

From a clinical perspective, the findings support a multifaceted approach combining individualized alarm parameters, optimization of monitoring devices, structured protocols, continuing education, and organizational support. Recent systematic review evidence similarly supports multifaceted alarm management strategies, particularly education and alarm customization, while highlighting the moderate-to-high risk of bias among the available intervention studies [39]. In Donabedian’s framework, these interventions predominantly relate to the structure and process dimensions of quality, emphasizing that effective alarm management depends on both the conditions under which care is delivered and the practices used to manage monitoring [29].

In practice, alarm parameters should be reviewed according to patients’ physiological status, while measures such as appropriate skin preparation, electrode replacement, equipment verification, and regular alarm checks may reduce nonactionable alarms [22,23]. Education, audit and feedback, multidisciplinary governance, and periodic protocol review may further support implementation and sustainability [12,16,31,33]. However, these recommendations should be interpreted primarily as strategies for improving alarm management processes, because direct evidence of improved patient outcomes remains limited.

Future research should prioritize prospective multicenter studies and, where feasible, randomized controlled trials using standardized outcome measures to evaluate the effectiveness and generalizability of alarm management interventions across ICU settings. Implementation research is also needed to examine the feasibility, sustainability, and contextual factors influencing the integration of evidence-based alarm management strategies into routine clinical practice. Future studies should clearly distinguish process outcomes, such as alarm frequency, alarm fatigue, and response practices, from direct patient-level clinical outcomes. In addition, emerging technologies, including artificial intelligence and clinical decision support systems, require prospective evaluation to determine their effects on alarm relevance, nurses’ workload and decision-making, and patient-level outcomes before widespread implementation.

### 4.7. Strengths and Limitations

This review has several methodological strengths. First, the inclusion of studies employing diverse methodological designs, including qualitative, quantitative, observational, quasi-experimental, quality improvement, and evidence synthesis studies, provided a comprehensive understanding of clinical alarm management in ICUs from clinical, organizational, technological, and educational perspectives. This methodological diversity is consistent with the purpose of integrative reviews, allowing evidence from different research approaches to be synthesized into a broader understanding of complex healthcare phenomena. A further strength is the rigorous methodological approach adopted throughout the review. The review followed the methodological framework proposed by Whittemore and Knafl [17], was reported in accordance with the PRISMA 2020 recommendations, and its protocol was prospectively registered with the OSF, thereby enhancing methodological transparency and reducing the risk of reporting bias. In addition, the methodological quality of the included studies was systematically assessed using the appropriate JBI Critical Appraisal Tools for each study design, supporting a more critical interpretation of the evidence synthesis. Another important contribution of this review is the identification and organization of nursing interventions into five thematic domains, providing a structured synthesis of the available evidence and facilitating the translation of research findings into clinical practice. This thematic organization may support the development of institutional alarm management programs and guide future research in this field.

Despite these strengths, several limitations should be acknowledged. Restricting the search to studies published between 2020 and 2025 ensured that the review reflected current clinical practice and technological developments but may have resulted in the exclusion of earlier studies that continue to be relevant to alarm management. Furthermore, limiting the review to publications in English, Portuguese, and Spanish may have introduced language bias. The evidence included in this review was characterized by considerable methodological heterogeneity, encompassing different study designs, intensive care settings, patient populations, outcome measures, and intervention characteristics. In addition, some included studies were not conducted exclusively in ICU settings, introducing contextual heterogeneity that should be considered when interpreting the applicability of the findings to ICU settings. Consequently, direct comparison between studies was challenging, and quantitative synthesis through meta-analysis was not appropriate. Moreover, most included studies consisted of quality improvement projects and observational research, with relatively few quasi-experimental studies and no large multicenter randomized trials. Therefore, although the findings consistently support multifaceted alarm management strategies, the overall strength of the evidence remains limited by the methodological characteristics of the available literature. Furthermore, most included studies evaluated alarm frequency, alarm fatigue, nurses’ knowledge or competence, and other process-related outcomes rather than direct patient-level clinical outcomes. Consequently, the findings should not be interpreted as evidence that the identified interventions directly improve patient safety or patient outcomes.

Finally, the relatively small number of included studies may limit the generalizability of the findings, and publication bias cannot be excluded, as studies reporting positive outcomes are more likely to be published than those with neutral or negative findings. Despite these limitations, this review provides an up-to-date synthesis of the available evidence, identifies consistent patterns across different methodological approaches, and highlights priorities for future research aimed at improving alarm management and patient safety in ICUs.

## 5. Conclusions

Effective clinical alarm management in ICUs requires an integrated approach combining individualized alarm parameters, optimization of monitoring devices, structured organizational protocols, continuing professional education, and attention to human and organizational factors. Across the nine included studies, these strategies were primarily associated with reductions in alarm burden and alarm fatigue and with improvements in nurses’ knowledge and competence and alarm management practices. However, direct patient-level clinical outcomes were rarely assessed; therefore, the findings should not be interpreted as demonstrating direct improvements in patient safety or patient outcomes.

The findings support the implementation of multifaceted alarm management strategies adapted to clinical and organizational contexts. Future research should prioritize prospective multicenter studies using standardized outcome measures and should distinguish process-related measures from direct patient-level clinical outcomes. Further evaluation of emerging technologies, including artificial intelligence and clinical decision support systems, is also needed to determine their effects on alarm management, nurses’ workload, and patient outcomes.

## Figures and Tables

**Table 1:** Inclusion and exclusion criteria for study selection.

Criterion	Inclusion Criteria	Exclusion Criteria
Publication period	Studies published between 2020 and 2025	Studies published outside the defined period
Language	English, Portuguese, or Spanish	Studies published in other languages
Publication status and availability	Full-text, peer-reviewed studies	Studies for which the full text was unavailable; non-peer-reviewed publications
Context	Studies addressing clinical alarm management associated with continuous physiological monitoring in intensive or critical care settings	Studies unrelated to alarm management in intensive or critical care settings
Focus	Studies addressing nursing interventions or nursing practice related to clinical alarm management	Studies that did not involve nursing practice
Study design	Qualitative, quantitative, mixed-methods, quality improvement studies, and evidence syntheses (systematic, integrative, and scoping reviews)	Opinion papers, editorials, book chapters without empirical evidence, and conference abstracts
Evidence overlap	Evidence syntheses providing relevant evidence without substantial overlap with included primary studies	Scoping reviews substantially overlapping with primary studies included in the review
Gray literature	—	Theses, dissertations, clinical guidelines, institutional protocols, and other forms of gray literature

**Table 2:** Characteristics of the included studies (*n* = 9).

Reference	Country	Study Design	Setting/Sample	Study Aim
Gorisek et al. [22]	United States	Quality improvement project	Burn ICU	To assess the effectiveness of evidence-based interventions in reducing nonactionable alarms and alarm fatigue
Seifert et al. [23]	United States	Quality improvement project	Medical-surgical ICU (15 beds)	To assess the impact of an alarm management bundle on the frequency of physiological alarms and alarm fatigue
Bosma and Christopher [24]	United States	Quality improvement project	Surgical ICU; 115 nurses	To assess the effectiveness of implementing the CEASE bundle in improving alarm management competencies and reducing alarm fatigue
Clemenson and Tellson [25]	United States	Quasi-experimental study	Pediatric unit with continuous monitoring	To assess the safety of modifying heart rate and respiratory rate parameters to reduce nonactionable alarms
Jeong and Kim [26]	South Korea	Descriptive study	48 nurses from two ICUs	To investigate nurses’ perceptions, alarm fatigue, and alarm management practices
Karahan et al. [8]	Turkey	Cross-sectional descriptive study	592 nurses from ICUs and hospital wards	To assess the level of alarm fatigue and identify associated factors
Movahedi et al. [27]	Iran	Qualitative study	18 nurses from 12 ICUs	To explore the strategies used by nurses to manage alarm fatigue
Arkilic et al. [28]	United States	Quality improvement project	Cardiothoracic ICU (24 beds); nurses and critically ill patients	To assess the impact of monitor alarm parameter individualization and educational interventions on alarm frequency and nurses’ alarm management
Li et al. [15]	China	Integrative review	Studies of intelligent alarm management interventions in ICUs	To assess the effectiveness of intelligent technology-based interventions in reducing false alarms

**Table 3:** Synthesis of nursing interventions and factors associated with alarm management during continuous monitoring.

Reference	Country	Study Design	Intervention or Factor Assessed	Key Findings
**Domain 1—Individualization of alarm parameters**				
Clemenson and Tellson [25]	United States	Quasi-experimental study	Adjustment of heart rate and respiratory rate parameters by ±15%	A 43% reduction in nonactionable alarms without compromising patient safety
Movahedi et al. [27]	Iran	Qualitative study	Alarm individualization and early identification of alarm causes	Participants identified these approaches as strategies for reducing unnecessary alarms and alarm fatigue
Arkilic et al. [28]	United States	Quality improvement project	Individualization of monitor parameters and nurse training	Alarm parameter individualization contributed to improved alarm management and greater awareness among healthcare professionals
**Domain 2—Nurse education and training**				
Gorisek et al. [22]	United States	Quality improvement project	Modular educational program on alarm safety and management	Sustained reduction in alarm frequency following the educational intervention
Bosma and Christopher [24]	United States	Quality improvement project	Training in alarm management associated with the CEASE bundle	Improved competencies and a significant reduction in alarm fatigue
Arkilic et al. [28]	United States	Quality improvement project	Educational sessions, informational flyers, and a training video on alarm management	Improved nurses’ competencies in alarm parameter individualization and response
**Domain 3—Structured alarm management protocols and strategies**				
Seifert et al. [23]	United States	Quality improvement project	Alarm management bundle (electrode replacement, skin preparation, and alarm individualization)	Reduction in the frequency of physiological alarms
Bosma and Christopher [24]	United States	Quality improvement project	Implementation of the CEASE bundle	Improved alarm management practices and reduced alarm fatigue
Li et al. [15]	China	Integrative review	Implementation of structured protocols and alarm management algorithms	The reviewed studies reported consistent improvements following the implementation of structured alarm management systems
**Domain 4—Optimization of monitoring devices and sensors**				
Gorisek et al. [22]	United States	Quality improvement project	Daily electrode replacement and improved sensor placement	Sustained reduction in nonactionable alarms
Seifert et al. [23]	United States	Quality improvement project	Standardization of sensor use and skin preparation	Improved monitoring quality and reduced alarm frequency
Bosma and Christopher [24]	United States	Quality improvement project	Appropriate skin preparation and regular electrode replacement	Significant reduction in technical alarms
Li et al. [15]	China	Integrative review	Use of intelligent devices and systems for alarm monitoring and notification	The review reported improvements in clinical response and efficiency through the use of intelligent systems and wearable devices
**Domain 5—Human and organizational factors affecting alarm management**				
Jeong and Kim [26]	South Korea	Descriptive study	Assessment of nurses’ perceptions and practices regarding clinical alarms	Identification of moderate to high levels of alarm fatigue
Karahan et al. [8]	Turkey	Descriptive study	Analysis of alarm fatigue among nurses	False alarms were identified as the main cause of alarm fatigue
Movahedi et al. [27]	Iran	Qualitative study	Smart care strategies, including teamwork and environmental organization	Participants reported that organizational strategies could reduce the impact of alarm fatigue

## Data Availability

No new data were created or analyzed in this study. Data sharing is not applicable to this article.

## Abbreviations

The following abbreviations are used in this manuscript:

CEASE	Communication, Electrodes, Appropriateness, Setup, and Education
ICU	Intensive care unit
JBI	Joanna Briggs Institute
MeSH	Medical Subject Headings
OSF	Open Science Framework
PRISMA	Preferred Reporting Items for Systematic Reviews and Meta-Analyses
WHO	World Health Organization

## Appendix A

**Table A1:** Item-level JBI critical appraisal of the included studies.

ID/Reference	JBI a Used	Q1	Q2	Q3	Q4	Q5	Q6	Q7	Q8	Q9	Q10	Q11
A1 Arkilic et al. [28]	Quasi-Experimental	Y	N	Y	N	N	—	Y	N	Y	—	—
A2 Bosma and Christopher [24]	Quasi-Experimental	Y	Y	Y	N	N	U	Y	Y	Y	—	—
A3 Clemenson and Tellson [25]	Quasi-Experimental	Y	Y	Y	N	N	—	Y	Y	Y	—	—
A4 Gorisek et al. [22]	Quasi-Experimental	Y	U	Y	N	Y	—	Y	Y	Y	—	—
A5 Jeong and Kim [26]	Analytical Cross-Sectional	Y	Y	Y	U	N	N	Y	Y	—	—	—
A6 Karahan et al. [8]	Analytical Cross-Sectional	Y	Y	U	U	Y	N	U	Y	—	—	—
A7 Li et al. [15]	Systematic Reviews and Research Syntheses	Y	Y	Y	Y	Y	U	U	Y	N	Y	Y
A8 Movahedi et al. [27]	Qualitative Research	U	Y	Y	Y	Y	N	N	Y	Y	Y	—
A9 Seifert et al. [23]	Quasi-Experimental	Y	Y	Y	N	N	U	Y	Y	Y	—	—

Abbreviations: Y, Yes; N, No; U, Unclear; —, item not included in the corresponding JBI Critical Appraisal Tool; JBI, Joanna Briggs Institute; Q, critical appraisal item. Note: Methodological appraisal results are presented at the item level using the response categories of the corresponding JBI Critical Appraisal Tools. Because different JBI tools were applied according to study design, Q1–Q11 correspond to the items of the specific JBI tool indicated for each study and should not be interpreted as equivalent across different appraisal tools. The appraisal findings were used to support the critical interpretation of the evidence and did not determine study inclusion or exclusion.
